# Kinetics of antifungal activity of home-generated ozonated water on *Candida albicans*

**DOI:** 10.18502/cmm.4.2.67

**Published:** 2018-06

**Authors:** Amirtaher Mirmortazavi, Hamidreza Rajati Haghi, Abdolmajid Fata, Hossein Zarrinfar, Hossein Bagheri, Amirhossein Mehranfard

**Affiliations:** 1 Department of Prosthodontics, School of Dentistry, Mashhad University of Medical Sciences, Mashhad, Iran; 2 Cutaneous Leishmaniasis Research Center, Mashhad University of Medical Sciences, Mashhad, Iran; 3 Department of Parasitology and Mycology, School of Medicine, Mashhad University of Medical Sciences, Mashhad, Iran; 4 Allergy Research Center, Mashhad University of Medical Sciences, Mashhad, Iran; 5 Dental Materials Research Center, Department of Operative Dentistry, School of Dentistry, Mashhad University of Medical Sciences, Mashhad, Iran

**Keywords:** Antifungal, Candida, Denture, Ozonated water, Stomatitis

## Abstract

**Background and Purpose:**

*Candida*-associated denture stomatitis is one of the most common forms of oral candidiasis among denture wearers. Regarding this, the aim of the present study was to evaluate the antifungal effects of home-generated ozonated water on the adhesion of the C.* albicans* attached to the surface of the denture base acrylic resins.

**Materials and Methods:**

For the purpose of the study, different concentrations of C. albicans were added to the tubes containing acrylic resin blocks, and then incubated for 2 h at 35°C. The samples were assigned into three groups, each of which contained 42 samples, including normal saline (NS) solution as the negative control, nystatin (N) solution as the positive control, and ozonated water as the test group. The samples were washed and placed in an ultrasonic bath. Subsequently, the saline solution was **cultured on Sabouraud dextrose agar. **The concentrations of *Candida *were evaluated during the contact times.

**Results:**

The test group (i.e., ozonated water) with 114 colony-forming units (CFU) showed a significant reduction of ***Candida* colonies,** compared to the NS group with 2,172 CFU**.** The 120- and 1-minute incubation with ozonated water showed the highest and lowest effects on the viability of *Candida* adhered to the acrylic resin, respectively.

**Conclusion:**

Based on the findings, home-generated ozonated water can be applied to remove the *Candida* attached to the surface of the denture plates.

## Introduction

Use of dentures may alter the oral environment and provide a favorable condition for the proliferation and colonization of microorg-anisms, especially *Candida*-associated denture stomatitis [1]. This condition is characterized by the moderate to severe inflammation of the mucosa underneath the denture material. Some factors may influence the development and severity of denture stomatitis. These factors includes the extent of denture-base fit, patient’s age and systemic conditions, poor diet and hygiene, and salivary flow [2]. 

*Candida* species, especially *C. albicans*, have a high affinity to adhere to and colonize on the acrylic resin denture base material, particularly rough surfaces [3, 4]. Therefore, it is inevitable to control denture plaque to prevent the *Candida*-associated denture stomatitis. The microbial biofilms, especially those created by fungal agents, can be controlled by mechanical, chemical, or combined mechanochemical methods. Several chemicals are introduced for denture disinfection; however, they have their own drawbacks, such as high cost and exertion of deteriorative effects on the denture base material [5, 6]. 

Ozone (O3), an allotrope of oxygen, is recognized as a strong oxidative antimicrobial agent. Ozonated water (OW) has been considered as an effective disinfectant agent against oral pathogens, including *Candida* [7, 8]. Ozone is a versatile substance in dentistry [9] and can be used in caries control [10], root canal disinfection [11], avulsed teeth disinfection before re-implantation [12], surgery as a hemostatic agent, postsurgical wound healing [13], sterilization of implant and bone surface, and tissue regeneration stimulation at implantation site [14]. 

Ozone also has a high potential to be used as an antimicrobial agent in endodontics. Based on the evidence, ozonation can be beneficial if the ozone is utilized with adequate concentration, suitable time, and correct method for reaching root canals when the traditional cleansing, shaping, and washing of canals are completed. The potentiality of ozone, OW, and ozonated oil to be used in endodontic therapy has been frequently reported in the literature [15]. 

It has been shown that OW can be mutagenic if used for a long period and in high concentrations [7]. Home ozone generators are portable, inexpensive, and user-friendly devices for producing ozone gas for home use, such as disinfection of water, vegetables, or air. However, the capacity of home ozone generators is lower than that of the industrial and medical types. If the OW produced by home ozone generators shows antifungal activity against *Candida*, it can be introduced as an inexpensive and easy method for denture cleaning/disinfection to prevent the denture stomatitis. With this background in mind, the present study was conducted to evaluate the antifungal properties of OW produced by a commercial-grade ozone generator. 

## Materials and Methods

***Fabrication of acrylic resin blocks***

The bar-shape acrylic resin specimens (25×10×3 mm) were fabricated using a conventional flasking and pressure-pack technique [7]. Silicon-made patterns having the mentioned dimensions were embedded in type-III dental stone in a brass flask. The flasks were separated by the stone set, and the silicon molds were removed. Subsequently, the spaces were filled with a commercial heat-curing acrylic resin denture base material (powder/liquid: 3/1 v/v; Marlik, Marlik Medical Industries Co., Tehran, Iran). 

The polymerization was accomplished in boiling water for 20 min based on the manufacturer’s instruction and remained in water at ambient temperature for 24 h. The flask parts were separated, and the specimens were removed, cleaned from remaining stone, and then well-polished. Each specimen was washed vigorously under tap water, put in a test tube, and finally autoclaved at 121°C for 15 min. 

***Fungal colonization and*** ***examination of antimicrobial activity***

*C. albicans* strain PTCC 5027 was plated on Sabouraud dextrose agar (SDA) (Merck, Merck KGaA, Darmstadt, Germany) to obtain fresh colonies. After a 48-hour incubation at 35°C, a suspension of 2×10^6 ^colony-forming unit (CFU) /ml was prepared using a Neubauer chamber. This suspension was considered as the primary *Candida *colony concentration. Other different concentrations of C. albicans  (2×10^5^, 2×10^4^, and 2×10^3^ CFU/ml) were prepared from the stock suspension via serial dilution method [16, 17]. 

In the next stage, each tube containing the specimen block was sealed and incubated for 2 h at 35°C. After the incubation, the blocks were washed three times with saline solution to remove the unattached *Candida*. Then, the samples were assigned into three groups, each of which containing 42 samples. Each group was further divided into seven sub-groups (n=6) to examine the effect of time on the antifungal activity. The study groups were as follows:

- Group of normal saline: normal saline solution was used as a negative control. All tubes were filled with 5 ml saline solution.
- Group of nystatin: nystatin solution (10.000 U/mL; Emad, Emad Darman Pars Co., Saveh, Iran) was used as a positive control (according to several reports) [18, 19].
- Group of OW: OW was used as the test group. The OW was prepared by a home air ozone generator (Sterile Air, ARDA, Tehran, Iran). The output tube was placed in a glass beaker containing 1.5 L deionized distilled water for 2 h.

After various periods of time (i.e., 1, 5, 10, 20, 40, 60, and 120 min), the content of the tubes in each group was discharged, and the specimens were washed vigorously; subsequently, 5 ml fresh saline solution was added to each tube. All tubes were placed in an ultrasonic bath (DT 510, Bandelin Electronic, Germany) containing 30°C distilled water for 30 min to detach the *Candida* from the block specimens [20]. Then, from each tube, a volume of 10 µL saline was added to petri dishes containing SDA medium and incubated for 48 h at 35°C. Finally, the number of *Candida *colonies was counted in each group. 

***Statistical analysis***

The relationship between the mean colony count in each group and time was estimated using a non-linear regression model. All statistical procedures were performed in SPSS software (version 16). P-value less than 0.05 was considered statistically significant. 

## Results and Discussion

Table 1 shows the descriptive results for the mean colony count of *Candida* at different incubation times in each group. According to the results, *Candida* CFU had a positive relationship with the number of colonies attached to the surface of the specimens. Regardless of the primary *Candida* CFU, no fungal colony was grown in the presence of nystatin. Furthermore, *Candida* colonies in the normal saline group demonstrated no significant changes. 

**Figure 1:**
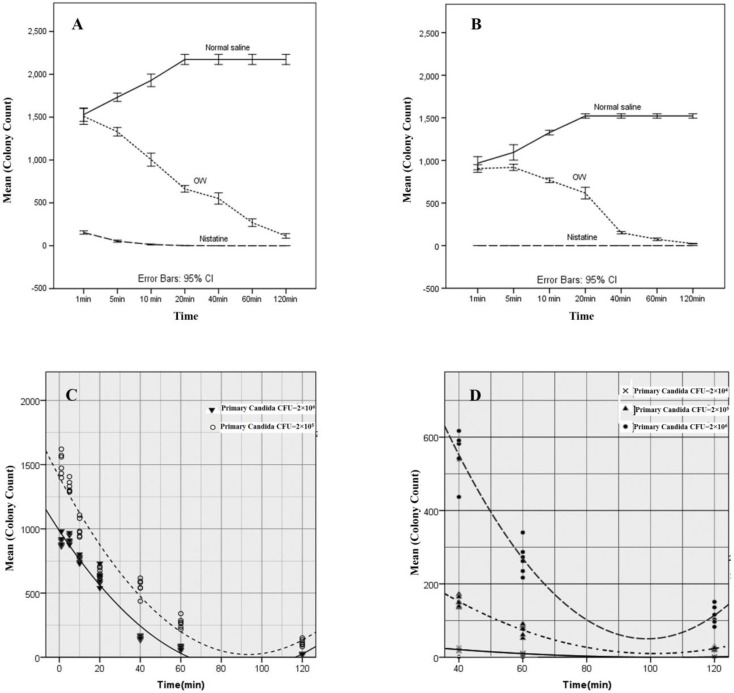
Colonization of *Candida albicans* on the surface of acrylic resin denture base material blocks in the presence of normal saline, nystatin, and ozonated water (A and B), kinetics of anti-fungal effect of ozonated water for different primary *Candida* CFUs (C and D)

The OW group showed an inhibitory effect on the growth and colonization of *C. albicans*; therefore, the null hypothesis was rejected. In addition, the *Candida* CFU demonstrated the non-linear goodness of fit (r^2^>0.93, *P<0.001*) to the time (Figure 1C) in these groups. Moreover, the changes in the mean *Candida* CFU showed a negative non-linear quadratic relationship with time from 40 to 120 min (r^2^=0.88, *P<0.001*) (Figure 1D). 

In *Candida* CFU of 2×10^5^, the mean *Candida* colony counts diminished between 40 and 60 min, while in *Candida* CFU of 2×10^6^, it took about 80 min to inhibit the colony growth. In lower suspensions of *Candida* CFU (2×10^3^), it was observed that the colony growth was almost inhibited after 40 min (Table 1 and Figure 1).

**Table 1:** Mean colony count of *Candida *following the different incubation times in each group

**Group**	**Primary ** ***Candida*** ** CFU** ^*^ ** /ml**	***Candida*** ** colony counts** **Mean (SD)**						
		**1 min**	**5 min**	**10 min**	**20 min**	**40 min**	**60 min**	**120 min**
Normal saline	2×10^6^	1529.67 (751.15)	1732.17 (47.03)	1928.83 (69.92)	2172.83 (55.90)	2172.83 (55.90)	2172.83 (55.90)	2172.83 (55.90)
	2×10^5^	967.83 (73.50)	1094.50 (86.23)	1326.33 (25.92)	1521.83 (24.56)	1521.83 (24.56)	1521.83 (24.56)	1521.83 (24.56)
Nystatin	2×10^6^	153.83 (18.44)	52.83 (11.62)	14.50 (6.41)	1.17 (1.47)	0	0	0
	2×10^5^	0	0	0	0	0	0	0
Ozonated water	2×10^6^	1509.17 (88.18)	1329.83 (46.96)	1004.00 (72.51)	663.17 (36.84)	551.33 (63.46)	269.00 (43.10)	114.33 (25.41)
	2×10^5^	904.67 (43.24)	918.33 (34.65)	766.83 (26.08)	616.33 (65.20)	152.00 (14.17)	74.33 (14.85)	23.83 (3.60)
	2×10^4^	-	-	-	-	21.00 (4.58)	0.33 (0.57)	0
	2×10^3^	-	-	-	-	2.3 (1.52)	10.00 (2.00)	0

* CFU: Colony-forming unit

The aim of this study was to evaluate the antifungal activity of the ozonated water solution produced by a home ozone/water generator as a simple and inexpensive method for the disinfection of denture base acrylic resin. The results showed that the application of home-generated OW was effective in the removal of *Candida* attached to the surface of the denture plates.

Salerano *et al*. reviewed the *Candida*-associated denture stomatitis phenomena and recommended the correct oral hygiene for the control and prophylaxis of the *Candida*-associated denture stomatitis [5]. Among *Candida* species, *C. albicans* is reported as the most common causative agent of different *Candida* infections, especially for developing the denture stomatitis [5, 21-25].

According to the literature, the growth of *Candida*, especially *C. albicans,* on the denture surface is affected by several factors. These factors include denture roughness [4], salivary flow and proteins [1], oral cavity pH [1], permeability of denture base material [26], oral hygiene, and systemic conditions, such as diabetes mellitus and xerostomia [27]. Various disinfection methods, including chemical agents or microwave irradiation, have been evaluated to remove the yeast plaques on the surfaces of the denture disinfection. Nonetheless, these methods have their own drawbacks, such as costliness and exertion of deteriorative effects on the denture base material [22, 28].

In the current study, the acrylic resin blocks were washed and moved to the ultrasonic device after certain periods of incubation in *Candida* suspension to detach the colonies attached to the material surface. Therefore, the number of isolated colonies at each time represented the *Candida* population survived on the surface of denture base material in the presence of OW. The results of our study showed that the home-generated OW could inhibit the growth of *C. albicans* on the denture base material. 

Previous studies have also shown the anti-microbial activity of OW against *C. albicans* in root canal system and acrylic denture base material [7]. Cardoso *et al*. [28] reported that OW decreased the number of *C. albicans* colony immediately after exposure. Arita *et al*. [7] showed that the number of *C. albicans* significantly decreased during the first 10 min and approached to zero after 30 min of the immersion of acrylic denture base material in OW. On the other hand, it is indicated that the poorer denture hygiene is associated with more oral colonization of *Candida* [29]. High levels of opportunistic microorganisms in the saliva have been recognized as an important predisposing factor for *Candida*-associated stomatitis [30]. 

In this study, different concentrations of *Candida* suspension (denoted as the primary *Candida* CFU), representing the patient’s oral hygiene, were used to evaluate the effect of infection load on the disinfection strength of OW. Based on the findings of the present study, higher concentrations of *Candida* can affect the colonies number growing on the denture base material. The relatively high inhibitory effect was achieved according to different primary *Candida* CFUs.

 As a result, the use of OW method could rapidly decrease the *Candida* CFU during the first 20 min of storage. This value even reached to zero in lower *Candida* CFUs after 40 min. However, in higher *Candida* CFUs, it was able to reach to zero after 120 min. Therefore, the time recommended for the immersion of denture in OW depends on the patient’s oral hygiene or other factors [5, 31, 32] affecting the load of infection. 

One of the limitations of this study was the sole examination of *C. albicans*; however, this species is most commonly associated with denture stomatitis among the *Candida* species. Although home ozone generators have a lower capacity for ozone production for antifungal applications than industrial devices [7, 28], they are accessible and effective. 

## Conclusion

As the findings of the present study indicated, the home-generated OW could inhibit *Candida* growth and colonization on the acrylic denture base material. Consequently, the home ozone/water generators can be used as inexpensive, portable, and effective storage medium for the disinfection of dentures, especially in high-risk patients. 

## References

[1] Webb BC, Thomas CJ, Willcox MD, Harty DW, Knox KW (1998). Candida-associated denture stomatitis Aetiology and management: a review Part 2 Oral diseases caused by Candida species. Aust Dent J.

[2] Mandali G, Sener ID, Turker SB, Ülgen H (2011). Factors affecting the distribution and prevalence of oral mucosal lesions in complete denture wearers. Gerodontology.

[3] Park SE, Periathamby AR, Loza JC (2003). Effect of surface‐charged poly (methyl methacrylate) on the adhesion of Candida albicans. J Prosthodont.

[4] Verran J, Maryan CJ (1997). Retention of Candida albicans on acrylic resin and silicone of different surface topography. J Prosthet Dent.

[5] Salerno C, Pascale M, Contaldo M, Esposito V, Busciolano M, Milillo L (2011). Candida-associated denture stomatitis. Med Oral Patol Oral Cir Bucal.

[6] Dantas AP, Consani RL, Sardi JC, Mesquita MF, Silva MC, Sinhoreti MA (2014). Biofilm formation in denture base acrylic resins and disinfection method using microwave. J Res Pract Dent..

[7] Arita M, Nagayoshi M, Fukuizumi T, Okinaga T, Masumi S, Morikawa M (2005). Microbicidal efficacy of ozonated water against Candida albicans adhering to acrylic denture plates. Oral Microbiol Immunol.

[8] De Faria ID, Ueno M, Koga-Ito CY, Urruchi WI, Balducci I, Jorge AO (2016). Effects of ozonated water on Candida albicans oral isolates. Braz J Oral Sci.

[9] Gupta M (2012). Ozone: an emerging prospect in dentistry. Indian J Dental Sci.

[10] Baysan A, Whiley R, Lynch E (2000). Antimicrobial effect of a novel ozone–generating device on micro–organisms associated with primary root carious lesions in vitro. Caries Res.

[11] Azarpazhooh A, Limeback H (2008). The application of ozone in dentistry: a systematic review of literature. J Dent.

[12] Ebensberger U, Pohl Y, Filippi A (2002). PCNA‐expression of cementoblasts and fibroblasts on the root surface after extraoral rinsing for decontamination. Dental Traumatol.

[13] Stübinger S, Sader R, Filippi A (2006). The use of ozone in dentistry and maxillofacial surgery: a review. Quintessence Int.

[14] Pattanaik B, Jetwa D, Pattanaik S, Manglekar S, Naitam DN, Dani A (2011). Ozone therapy in dentistry: a literature review. J Interdiscipl Dent.

[15] Estrela C, Estrela C, Decurcio DA, Hollanda AC, Silva JA (2007). Antimicrobial efficacy of ozonated water, gaseous ozone, sodium hypochlorite and chlorhexidine in infected human root canals. Int Endod J.

[16] Katiraee F, Eidi S, Bahonar AR, Zarrinfar H, Khosravi AR (2008). Comparision of MICs of some Iranian herbal essences against azole resistance and azole susceptible of Candida albicans. J Med Plants.

[17] Rodríguez-Tudela JL, Cuenca-Estrella M, Díaz-Guerra TM, Mellado E (2001). Standardization of antifungal susceptibility variables for a semiautomated methodology. J Clin Microbiol.

[18] Seleem D, Benso B, Noguti J, Pardi V, Murata RM (2016). In vitro and in vivo antifungal activity of lichochalcone-a against Candida albicans biofilms. PLoS One.

[19] Melkoumov A, Goupil M, Louhichi F, Raymond M, de Repentigny L, Leclair G (2013). Nystatin nanosizing enhances in vitro and in vivo antifungal activity against Candida albicans. J Antimicrob Chemother.

[20] Cruz PC, Andrade IM, Peracini A, Souza-Gugelmin MC, Silva-Lovato CH, Souza RF (2011). The effectiveness of chemical denture cleansers and ultrasonic device in biofilm removal from complete dentures. J Appl Oral Sci.

[21] Zarrinfar H, Kaboli S, Dolatabadi S, Mohammadi R (2016). Rapid detection of Candida species in bronchoalveolar lavage fluid from patients with pulmonary symptoms. Braz J Microbiol.

[22] Webb BC, Thomas CJ, Willcox MD, Harty DW, Knox KW (1998). Candida-associated denture stomatitis Aetiology and management: a review Part 1 Factors influencing distribution of Candida species in the oral cavity. Aust Dent J.

[23] Esmailzadeh A, Zarrinfar H, Fata A, Sen T (2018). High prevalence of candiduria due to non‐albicans Candida species among diabetic patients: a matter of concern?. J Clin Lab Anal.

[24] Camargo GA, Abreu MG, Cordeiro Rdos S, Wenderoscky Lde F, Duque C (2016). Prevalence of periodontopathogens and Candida spp in smokers after nonsurgical periodontal therapy - a pilot study. Braz Oral Res.

[25] Alizadeh M, Kolecka A, Boekhout T, Zarrinfar H, Ghanbari Nahzag MA, Badiee P (2017). Identification of Candida species isolated from vulvovaginitis using matrix assisted laser desorption ionization-time of flight mass spectrometry. Curr Med Mycol.

[26] Ferreira MÁ, Pereira-Cenci T, Rodrigues de Vasconcelos LM, Rodrigues-Garcia RC, Del Bel Cury AA (2009). Efficacy of denture cleansers on denture liners contaminated with Candida species. Clin Oral Investig.

[27] Campisi G, Panzarella V, Matranga D, Calvino F, Pizzo G, Lo Muzio L (2008). Risk factors of oral candidosis: a twofold approach of study by fuzzy logic and traditional statistic. Arch Oral Biol.

[28] Cardoso MG, de Oliveira LD, Koga-Ito CY, Jorge AO (2008). Effectiveness of ozonated water on Candida albicans, Enterococcus faecalis, and endotoxins in root canals. Oral Surg Oral Med Oral Pathol Oral Radiol Endod.

[29] Darwazeh AM, Al-Refai S, Al-Mojaiwel S (2001). Isolation of Candida species from the oral cavity and fingertips of complete denture wearers. J Prosthet Dent.

[30] Pires FR, Santos EB, Bonan PR, De Almeida OP, Lopes MA (2002). Denture stomatitis and salivary Candida in Brazilian edentulous patients. J Oral Rehabil.

[31] Lotfi-Kamran MH, Jafari AA, Falah-Tafti A, Tavakoli E, Falahzadeh MH (2009). Candida colonization on the denture of diabetic and non-diabetic patients. Dent Res J.

[32] Figueiral MH, Fonseca P, Lopes MM, Pinto E, Pereira-Leite T, Sampaio-Maia B (2015). Effect of denture-related stomatitis fluconazole treatment on oral Candida albicans susceptibility profile and genotypic variability. Open Dent J..

